# Efficacy of a novel bivalent vaccine containing porcine circovirus type 2d and *Mycoplasma hyopneumoniae* against a dual PCV2d and *Mycoplasma hyopneumoniae* challenge

**DOI:** 10.3389/fvets.2023.1176091

**Published:** 2023-07-26

**Authors:** Sehyeong Ham, Jeongmin Suh, Taehwan Oh, Chonghan Kim, Byoung-Joo Seo, Chanhee Chae

**Affiliations:** ^1^Department of Veterinary Pathology, College of Veterinary Medicine, Seoul National University, Seoul, Republic of Korea; ^2^WOOGENE B&G CO., LTD., Seoul, Republic of Korea

**Keywords:** bivalent vaccine, enzootic pneumonia, *Mycoplasma hyopneumoniae*, porcine circovirus type 2d, porcine respiratory disease complex

## Abstract

**Background:**

Information on efficacy of a novel bivalent vaccine containing porcine circovirus type 2d (PCV2d) and *Mycoplasma hyopneumoniae*.

**Objective:**

To evaluate bivalent vaccine for efficacy under experimental conditions.

**Animals:**

Clinically healthy 35 weaned piglets at 18 days of age were used.

**Methods:**

A 2.0 mL dose of bivalent vaccine was administered intramuscularly to pigs at 21 days of age in accordance with the manufacturer’s instructions. The pigs were challenged at 42 days of age either intranasally with PCV2d, or intratracheally with *M. hyopneumoniae*, or with both.

**Results:**

Vaccinated-challenged pigs improved the growth performance compared to pigs that were unvaccinated and then, challenged. Vaccinated-challenged pigs elicited a significant amount of protective immunity for PCV2d-specific neutralizing antibodies and interferon-γ secreting cells (IFN-γ-SC) as well as for *M. hyopneumoniae*-specific IFN-γ-SC compared to unvaccinated/challenged pigs. Induction of systemic cellular and humoral immune responses from bivalent vaccination reduced the viral and mycoplasmal loads in the blood and larynx. Vaccination and challenge simultaneously reduced both lung and lymphoid lesion severity when compared to unvaccinated-challenged pigs.

**Discussion:**

The results of this study demonstrated that the evaluated bivalent PCV2d and *M. hyopneumoniae* vaccine was efficacious in protecting pigs from the most predominant PCV2d genotype in the field today, as evaluated with a dual PCV2d and *M. hyopneumoniae* challenge under experimental conditions.

## Introduction

Porcine circovirus 2 (PCV2) may best be described as a small but mighty virus. Postweaning multisystemic wasting syndrome (PMWS) was first reported in 1997 where the Western Canadian occurrence drew the attention of swine researchers and practitioners (1, 2). PCV2 is the essential infectious causative agent of porcine circovirus-associated diseases (PCVAD) and is one of the most devastating and economically damaging pig diseases in the world. PCVAD denotes the entire spectrum of diseases that are associated with PCV2 including PMWS, porcine respiratory disease complex (PRDC), porcine dermatitis and nephropathy syndrome (PDNS), reproductive failure, and enteric disease (3, 4).

*Mycoplasma hyopneumoniae* is the primary causative agent for enzootic pneumonia, a highly contagious respiratory disease in pigs (5, 6). The disease causes large economical losses due to reduced growth performance, reduced feed efficiency, increased days to market, increased susceptibility to other respiratory infections, and increased medication cost (5, 6).

The genetic gap between commercial PCV2 vaccines and the geographic prevalence of field viruses is increasing. All major commercially available PCV2 vaccines contain PCV2a and/or PCV2b while PCV2d is particularly predominant in the Asian and North American field (7–12). PCV2d has proven as more virulent than PCV2a and PCV2b in co-infection models (13–16). Moreover, PCV2d was repeatedly reported in association with perceived vaccine failures in several countries, indicating a “leaky” or imperfect vaccine situation (17–19). These field observations raise concern that commercially available PCV2a-based vaccines may not be fully capable of providing protection against the currently predominant PCV2d field genotype. Therefore, PCV2d-based vaccines may offer better protection against PCV2d infections compared to PCV2a-based vaccines. A new single-dose bivalent vaccine containing inactivated whole PCV2d and *M. hyopneumoniae* has recently come onto the market. The objective of this study was to determine the efficacy of a bivalent vaccine containing PCV2d and *M. hyopneumoniae* against a dual experimental PCV2d and *M. hyopneumoniae* challenge.

## Materials and methods

### Ethical statement

All of the methods were approved by the Seoul National University Institutional Animal Care and Use, and Ethics Committee (SNU-210308-2-1).

### Animals

Clinically healthy 35 weaned piglets at 18 days of age were selected from sows which were not immunized for either PCV2 or *M. hyopneumoniae*. The commercial farm was free of porcine reproductive and respiratory syndrome virus (PRRSV) and *M. hyopneumoniae* based on long-term serological and clinical test. Piglets were screened after arriving and results were seronegative for PCV2 (INgezim CIRCO IgG, Ingenasa, Madrid, Spain) and PRRSV (HerdChek PRRS X3 Ab test, IDEXX Laboratories Inc., Westbrook, ME, United States). Quantitative PCR was performed for PCV2 and PRRSV antigen in serum and tested as negative. Pigs were screened for *M. hyopneumoniae* and tested as seronegative by ELISA [*M. hyopneumoniae* (*M. hyo* Ab test, IDEXX Laboratories Inc.)] and negative by real-time PCR for *M. hyopneumoniae* in the larynx.

### Vaccine

A unique bivalent vaccine known as IMMUNIS® DMVac (WOOGENE B&G CO. Ltd., Seoul, Republic of Korea, http://www.woogenebng.com) is composed of the inactivated whole PCV2d strain SNUVR201901 (1 × 10^5^ TCID_50_/mL) and *M. hyopneumoniae* strain WGB-Mhp bacterin (OD 0.12 at 410 nm/mL). It was adjuvanted with carbopol (2.0 mg/mL) and saponine (2.0 μg/mL). IMMUNIS® DMVac is administered as one (2.0 mL) dose intramuscularly at 3 weeks of age and onward.

### Experimental design

There were seven groups, each consisting of five pigs (two were male and three were female), for a total of 35 pigs and each group was segregated into a different chamber (Table 1). Each room was of the same size and design and provided the pigs free access to drinking water and feed a corn-soybean diet. The diet was a commercially available weaner diet that was formulated to provide 3,000 kcal/kg.

**Table 1:** Experiment design with vaccination and challenge of porcine circovirus type 2d (PCV2d) and *Mycoplasma hyopneumoniae* (Mhp).

Group	Vaccination (days of age)	Challenge (days of age)	
		PCV2	Mhp
Vac/ChPM	21 (2 mL)	42	42
Vac/ChP	21 (2 mL)	42	-
Vac/ChM	21 (2 mL)	-	42
UnVac/ChPM	-	42	42
UnVac/ChP	-	42	-
UnVac/ChM	-	-	42
UnVac/UnCh	-	-	-

A 2.0 mL dose of bivalent vaccine containing PCV2d and *M. hyopneumoniae* (IMMUNIS® DMVac, Serial No: 23421D, Expiration date: 03-Feb-2023, WOOGENE B&G CO. LTD.) was administered intramuscularly into the neck muscles of pigs in the Vac/ChPM, Vac/ChP, and Vac/ChM groups at −21 days post challenge (dpc, 21 days of age). A 2.0 mL dose of phosphate buffered saline (PBS, 0.01 M, pH 7.4) was administered to pigs in the UnVac/ChPM, UnVac/ChP, UnVac/ChM, and UnVac/UnCh groups in the same manner as the vaccinates described above.

Pigs were challenged with PCV2d (SNUVR202003 strain, GenBank no. MZ440695, fifth passage in PCV-free PK-15 cell lines) in the Vac/ChPM, Vac/ChP, UnVac/ChPM, and UnVac/ChP groups at 0 dpc (42 days of age). Pigs were challenged with intranasally with PCV2d with a 3 mL inoculation containing 1.2 × 10^5^ (50% tissue culture infective dose/mL). An *M. hyopneumoniae* strain SNU98703, 20th passage, was grown 48–96 h in Friis mycoplasma broth in a rotary shaking incubator (37 C and 50 rpm) (20). An *M. hyopneumoniae* strain SNU98703, derived from field strain from 2010 in an outbreak in Seoul, Korea was used as challenge and was administered 5 h post-PCV2d challenge. This interval was implemented between the two challenges to avoid mixing the two pathogens, which could have resulted in a decrease in infectivity. Pigs were sedated and anesthetized by inoculating a mixture of 2.2 mg/kg xylazine hydrochloride (Rompun, Bayer Healthcare, Shawnee Mission, KS, United States), 2.2 mg/kg tiletamine hydrochloride, and 2.2 mg/kg zolazepam hydrochloride (Zoletil 50, Virbac, Carros, France) intramuscularly post-wait interval and immediately prior to *M. hyopneumoniae* challenge. 7 mL of *M. hyopneumoniae* strain SNU98703 culture medium containing 10^7^ color changing units/mL was inoculated intratracheally as previously described (21). Pigs in the Vac/ChP and UnVac/ChP groups only challenged with the PCV2d intranasally as described above, and pigs in the Vac/ChM and UnVac/ChM groups were only challenged with the same *M. hyopneumoniae* as explained above in the double challenged group sections.

Pigs were sedated at 21 dpc (63 days of age) regardless of treatment by an intravenous injection of sodium pentobarbital prior to euthanization by electrocution as previously described (22). Tissues such as lung, subinguinal lymph node, tonsil, liver, heart, and intestine were collected from each pig at necropsy then fixed in a 10% neutral buffered formalin solution, before they were embedded in paraffin.

### Sampling collection

Blood samples and laryngeal swabs were collected from all pigs at −21, 0, 7, 14, and 21 dpc as previous described (23).

### Clinical observation

Pigs were monitored daily and scored weekly for clinical signs by observers that were blinded to vaccination status. Scoring was defined according to the following scale: 0 (normal), 1 (rough haircoat), 2 (rough haircoat and mild dyspnea), 3 (mild dyspnea and abdominal breathing), 4 (moderate dyspnea and abdominal breathing), 5 (severe dyspnea and abdominal breathing), and 6 (death).

### Average daily weight gain

Throughout the study, pigs were weighed at −21 (21 days of age), 0 (42 days of age), and 21 (63 days of age) dpc. Upon conclusion of the study, an average daily weight gain (ADWG = grams/pig/day) was calculated over three time points: (i) between −21 and 0 dpc, (ii) between 0 and 21 dpc, and (iii) between −21 and 21 dpc. ADWG was calculated by dividing the difference between the initial and final weights at each of the three experimental stages by the number of days in the period. Data were obtained in a blinded manner.

### Quantification of PCV2d DNA

DNA was extracted from PCV2d serum samples with a commercial kit (QIAamp DNA Mini Kit, QIAGEN, Valencia, CA, United States). These extractions were then used to quantify the number of genomic DNA copies for both PCV2d (24). To construct a standard curve, real-time PCRd was performed in quadruplicate in 10-fold serial dilutions of the PCV2d DNA from PCV2d strain SNUVR202003, with concentrations ranging from 10^10^ to 10^2^ copies/mL. The detection limit of the assay was 1.2 × 10^2^ genomic copy numbers of PCV2d genotype (24). A negative control was included in each run using double distilled water as the template. Test was performed in duplicate. Data were obtained in a blinded manner.

### Quantification of *Mycoplasma hyopneumoniae* DNA

DNA was extracted from *M. hyopneumoniae* laryngeal swabs with a commercial kit (QIAamp DNA Mini Kit, QIAGEN, Valencia, CA, United States). These extractions were then used to quantify the number of genomic DNA copies for *M. hyopneumoniae* (25). Test was performed in duplicate. To construct a standard curve, real-time PCR was performed in quadruplicate in 10-fold serial dilution of chromosomal DNA from *M. hyopneumoniae* strain SNU98703, with concentrations ranging from 10 ng/μL to 1 fg/μL. One femtogram of chromosomal DNA from *M. hyopneumoniae* is approximately one genome equivalent (26). A negative control was included in each run using double distilled water as the template. Test was performed in duplicate. Data were obtained in a blinded manner.

### Enzyme-linked immunosorbent assay

Antibody presence to both PCV2 and *M. hyopneumoniae* were tested in serum samples using two commercially available enzyme-linked immunosorbent assay (ELISA) kits (INgezim CIRCO IgG, Ingenasa, Madrid, Spain and *M. hyo* Ab test, IDEXX Laboratories Inc. Westbrook, Maine, United States). Kits were used in accordance with the manufacturer’s instructions. PCV2 serum samples were considered positive for PCV2 antibodies if the optical density (OD) was ≥0.3 and positive for *M. hyopneumoniae* antibody if the sample-to-positive (S/P) ratio was ≥0.4, as outlined by manufacturer’s instructions. ELISA test was performed in duplicate. Data were obtained in a blinded manner.

### Serum neutralization test

PK-15 cells were planted into 96-well microtitration plates and used as an indicator for a serum viral neutralization assay (27, 28). Each serum samples were serially diluted 2-fold up to 1:512. An equal volume of each sample dilution was mixed with equal volume of PCV2d stock at the titer of 200 TCID_50_/0.1 mL. Thus, the lowest dilution contained 25% (1:1 dilution + equal volume of PCV2 stock), thereby the detection limit of the assay was 2.0 log_2_. A positive and negative serum control from previous study was included in each run (29). Serum neutralization test was performed in duplicate. Data were obtained in a blinded manner.

### Enzyme-linked immunospot

The numbers of PCV2d-and *M. hyopneumoniae*-specific interferon-γ secreting cells (IFN-γ-SC) were measured with an enzyme-linked immunospot (ELISpot) assay. The challenge strains for PCV2d and *M. hyopneumoniae* were used to stimulate peripheral blood mononuclear cells (PBMC), and results were reported as the number of IFN-γ-SC per million PBMC (24, 30). ELISpot assay was performed in duplicate. Data were obtained in a blinded manner.

### Pathology

Macroscopic lung lesion severity was scored by two veterinary pathologists at the Seoul National University in order to estimate the percentage of the lung affected by pneumonia (31). To achieve this, lung and sections were collected and examined, then scored. Scoring was based on the severity of peribronchiolar lymphoid tissue hyperplasia by mycoplasmal pneumonia lesions on a scale of 0–6 (32). Real-time PCR confirmed that the lesions were a result of mycoplasmal pneumonia as previously described (25). Lymphoid lesion severity was scored (0–5) based on lymphoid depletion and granulomatous inflammation (33). Data were obtained in a blinded manner.

### Immunohistochemistry

Porcine circovirus 2 immunohistochemistry was performed as previously described (34). Slides were prepared for immunohistochemistry morphometric analysis by sectioning off three cuts from three blocks of lymph node tissue per pig. Quantitative data was analyzed from the prepared immunohistochemistry slides using the NIH Image J 1.45 s Program.^1^ Ten fields were selected at random for PCV2 analysis, and the number of the positive cells per unit area (0.25 mm^2^) was determined as previously described (35) along with calculated mean values. Data were obtained in a blinded manner.

### Statistical analysis

For statistical processing, real-time PCR data and neutralizing antibody titers were converted into decimal logarithmic and binary logarithmic values, respectively. A normal distribution was determined with the Shapiro–Wilk on these data. Whether or not the groups had statistically significant differences between them at various timepoints was then determined by performing a one-way ANOVA. For the further evaluation, a *post hoc* test for a pairwise comparison with Tukey’s adjustment was conducted with a statistical significance result from a one-way ANOVA test. A Kruskal-Wallis test was additionally performed only in cases where the normality assumption was not met. Results which showed a statistical significance from the Kruskal-Wallis test were further evaluated with the Mann–Whitney test to compare the differences among the groups. Results were reported in *p* values and the values of *p* < 0.05 were considered significant.

## Results

### Respiratory clinical outcomes

**Figure 1:**
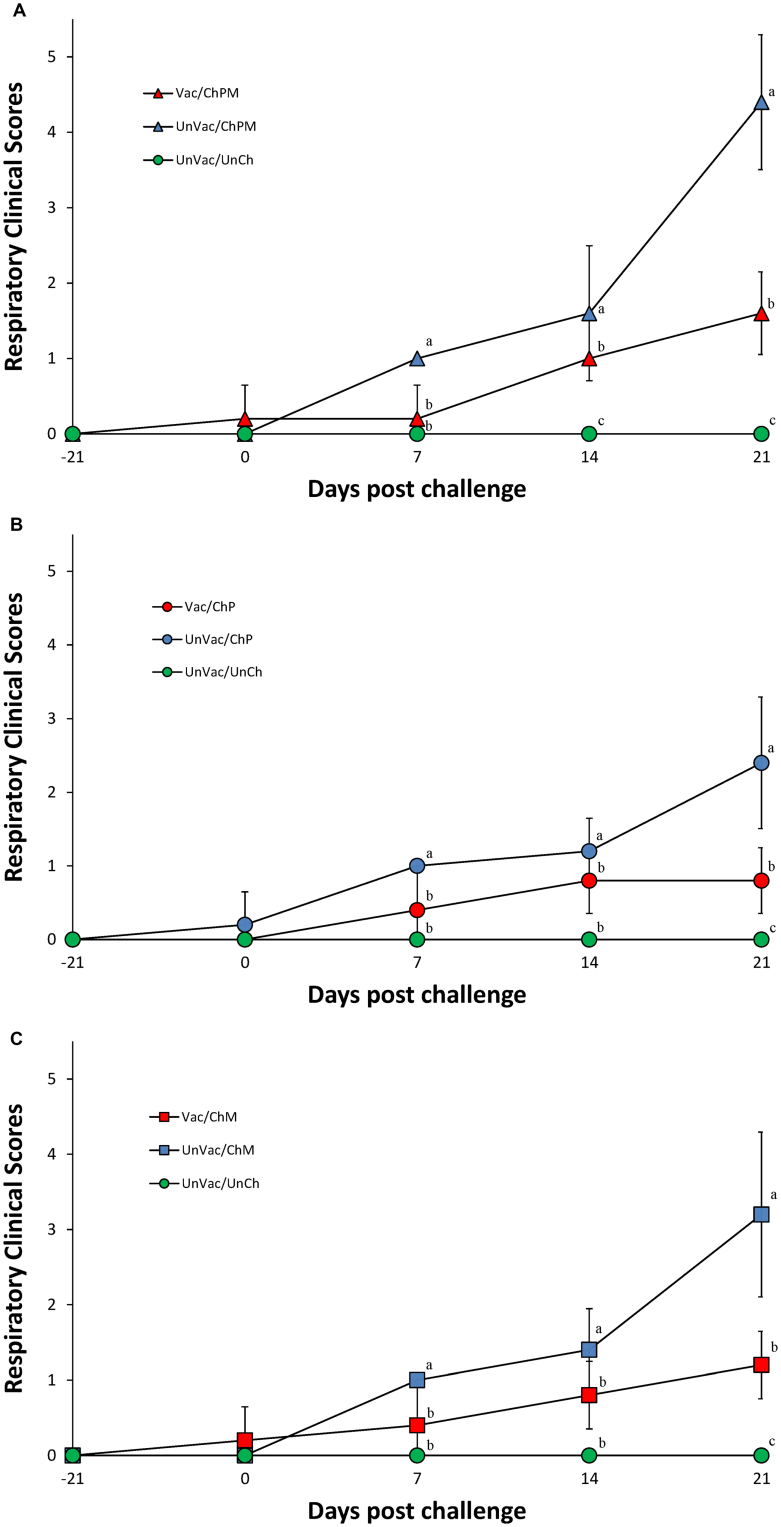
Mean values of respiratory clinical scores. Variation is expressed as the standard deviation. **(A)** Porcine circovirus type 2d (PCV2d)- and *Mycoplasma hyopneumoniae*-challenged groups. **(B)** PCV2d-challenged groups. **(C)** *Mycoplasma hyopneumoniae*-challenged groups. Different letters within a sampling point mean statistically significant differences (*p* < 0.05).

Within the dually challenged groups (PCV2d and *M. hyopneumoniae*), pigs in the Vac/ChPM group had significantly lower (*p* < 0.05) respiratory clinical scores than those in the UnVac/ChPM group at 7, 14, and 21 dpc (Figure 1A). A similar response was measured between the single PCV2d-challenged groups where pigs in the Vac/ChP group had significantly lower (*p* < 0.05) respiratory clinical scores than those in the UnVac/ChP group at 7 and 21 dpc (Figure 1B). Responses from sole-*M. hyopneumoniae* challenge pigs followed suite, where pigs in the Vac/ChM group were significantly lower (*p* < 0.05) in respiratory clinical scores at 7 and 21 dpc compared with pigs in the UnVac/ChM groups (Figure 1C). Respiratory clinical signs were not observed in the pigs in the UnVac/UnCh group throughout the entire experiment.

### Growth performance

**Figure 2:**
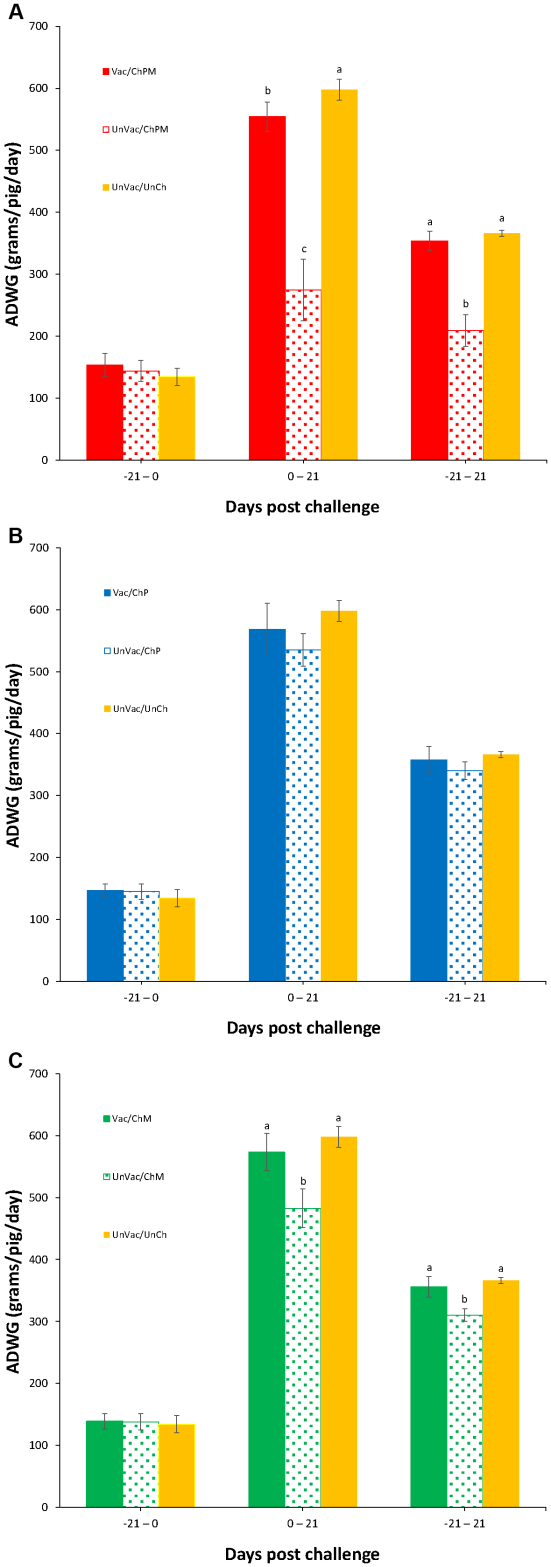
Mean values of average daily weight gain (ADWG). Variation is expressed as the standard deviation. **(A)** Porcine circovirus type 2d (PCV2d)- and *Mycoplasma hyopneumoniae*-challenged groups. **(B)** PCV2d-challenged groups. **(C)** *Mycoplasma hyopneumoniae*-challenged groups. Different letters within a sampling point mean statistically significant differences (*p* < 0.05).

The body weight of the pigs was evaluated at −21 and 0 dpc, where significant differences between the seven groups were not found. Pigs in the Vac/ChPM that received a dual PCV2d and *M. hyopneumoniae* challenge and UnVac/UnCh groups had significantly higher (*p* < 0.05) body weights at 21 dpc than the pigs in the UnVac/ChPM group. A significantly higher (*p* < 0.05) ADWG was measured in pigs in the Vac/ChPM and UnVac/UnCh groups compared to pigs in the UnVac/ChPM group between 0 and 21 dpc. Pigs that received a single *M. hyopneumoniae* challenge, such as the Vac/ChM and UnVac/UnCh groups had significantly higher (*p* < 0.05) body weights compared to pigs in the UnVac/ChM group. Pigs in the Vac/ChM and UnVac/UnCh groups had a significantly higher (*p* < 0.05) ADWG than pigs in the UnVac/ChM group between 0 and 21 and between 0 and 21 dpc (Figure 2).

### Genomic copies of PCV2d in serum

**Figure 3:**
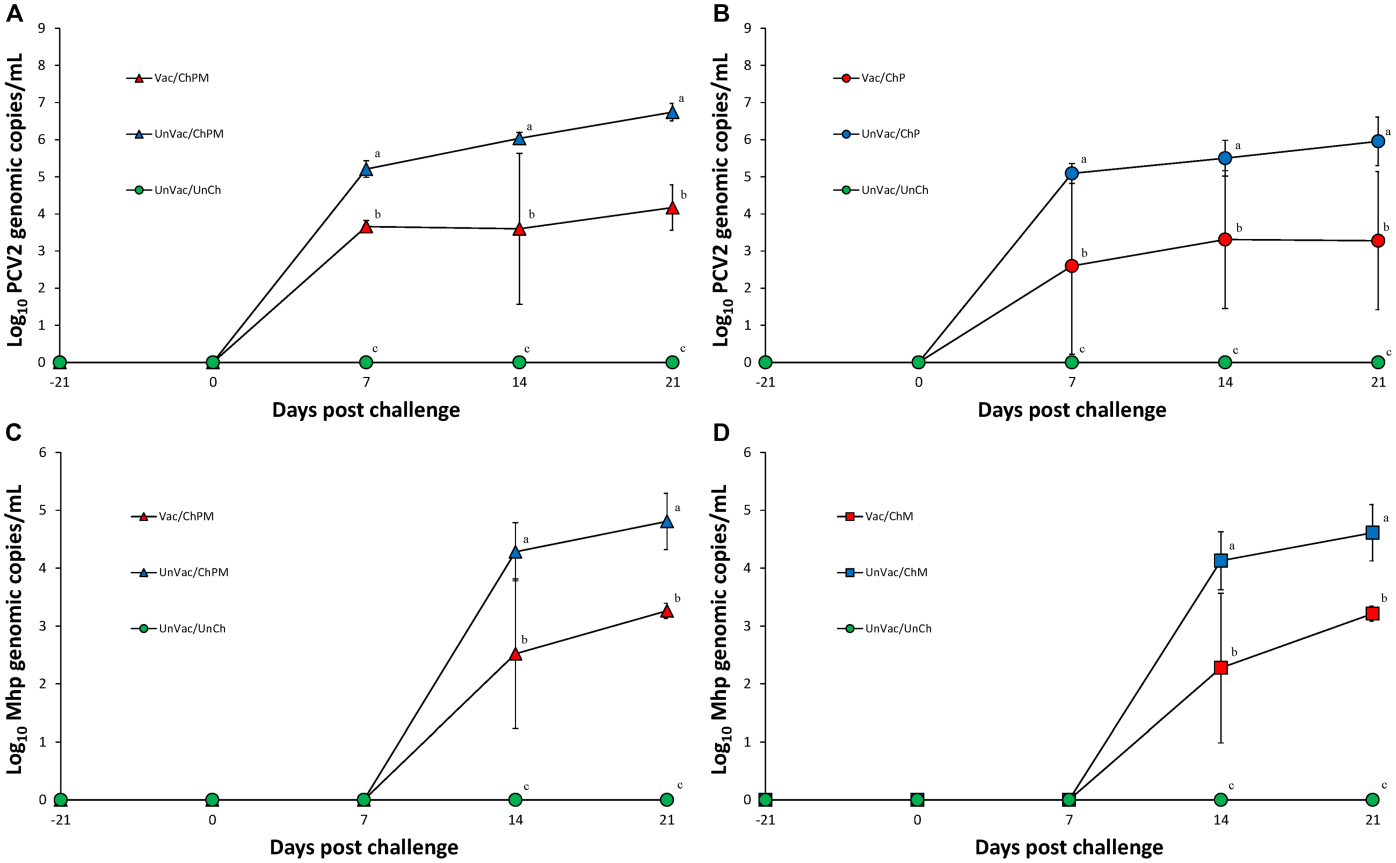
Mean values of the genomic copy numbers of PCV2d DNA in serum and *Mycoplasma hyopneumoniae* in larynx. Variation is expressed as the standard deviation. **(A)** Mean values of the genomic copy numbers of PCV2d DNA in PCV2d-and *M. hyopneumoniae*-challenged groups. **(B)** Mean values of the genomic copy numbers of PCV2d DNA in PCV2d-challenged groups. **(C)** Mean values of the genomic copy numbers of *M. hyopneumoniae* DNA in PCV2d-and *M. hyopneumoniae*-challenged groups. **(D)** Mean values of the genomic copy numbers of *M. hyopneumoniae* DNA in *M. hyopneumoniae*-challenged groups. Different letters within a sampling point mean statistically significant differences (*p* < 0.05).

Serum was evaluated and determined negative of PCV2d genomic copies at both the time of vaccination and the time of PCV2d challenge. Vaccination and challenge significantly (*p* < 0.05) reduced the amount of PCV2d genomic copies found in the serum of pigs from the Vac/ChPM and Vac/ChP groups at 7, 14, and 21 dpc compared to unvaccinated-challenged pigs from the UnVac/ChPM and UnVac/ChP groups (Figures 3A,B). Unvaccinated-unchallenged pigs remained free of PCV2d genomic copies as tested in the sera of the UnVac/UnCh group throughout the duration of the experiment.

### Genomic copies of *Mycoplasma hyopneumoniae* in larynx

Real-time PCR results for *M. hyopneumoniae* were negative in the larynx of any pigs at the time of both vaccination and *M. hyopneumoniae* challenge. Pigs evaluated at 14 and 21 dpc for the presence of *M. hyopneumoniae* genomic copies in the larynx where vaccination and challenge (Vac/ChPM and Vac/ChM groups) had significantly (*p* < 0.05) reduced these values compared to unvaccinated-challenged pigs (UnVac/ChPM and UnVac/ChM; Figures 3C,D). *Mycoplasma hyopneumoniae* was not detected in the larynx of unvaccinated-unchallenged negative control (UnVac/UnCh) group throughout the duration of the experiment using real-time PCR.

### Immunological responses against PCV2d

**Figure 4:**
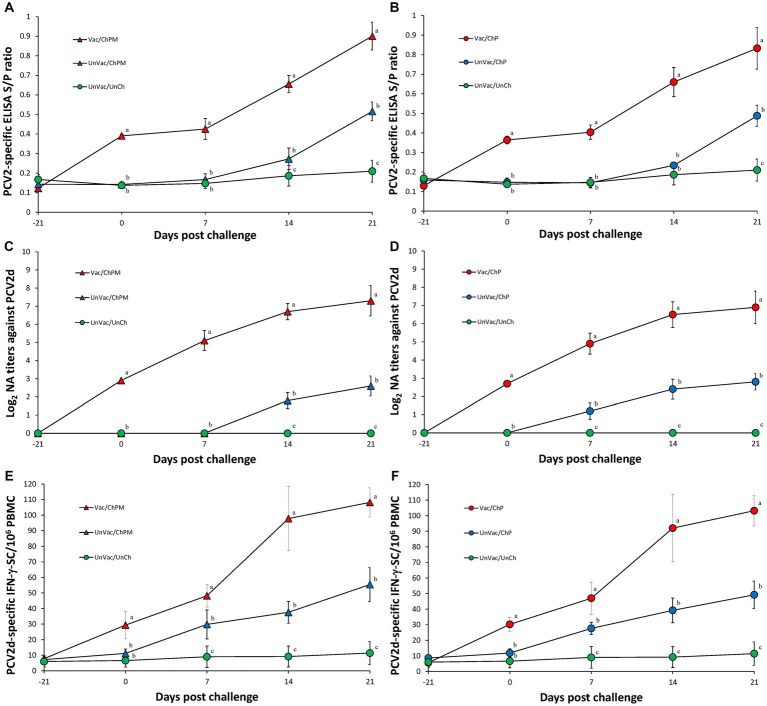
Immune responses against porcine circovirus type 2 (PCV2). **(A)** Mean values of the ELISA anti-PCV2 antibodies in PCV2d-and *Mycoplasma hyopneumoniae*-challenged groups. **(B)** Mean values of the ELISA anti-PCV2 antibodies in PCV2d-challenged groups. **(C)** Mean values of the neutralizing antibody (NA) titers in PCV2d-and *M. hyopneumoniae*-challenged groups. **(D)** Mean values of the NA titers in PCV2d-challenged groups. **(E)** Frequency of PCV2d-specific interferon-γ secreting cells (IFN-γ-SC) in PCV2d-and *M. hyopneumoniae*-challenged groups. **(F)** Mean values of the PCV2d-specific IFN-γ-SC levels in PCV2d-challenged groups. Different letters within a sampling point mean statistically significant differences (*p* < 0.05).

Vaccination and challenge (Vac/ChPM and Vac/ChP groups) resulted in significantly (*p* < 0.05) higher anti-PCV2 antibody levels, neutralizing anti-PCV2d antibody levels, and PCV2d-specific IFN-γ-SC levels compared to unvaccinated-challenged pigs (UnVac/ChPM and UnVac/ChP groups) at 0, 7, 14, and 21 dpc (Figure 4). Anti-PCV2 antibody, neutralizing anti-PCV2 antibody, and PCV2d-specific IFN-γ-SC were not detected in the unvaccinated-unchallenged pigs from the negative control (UnVac/UnCh) group throughout the entire experiment.

### Immunological responses against *Mycoplasma hyopneumoniae*

**Figure 5:**
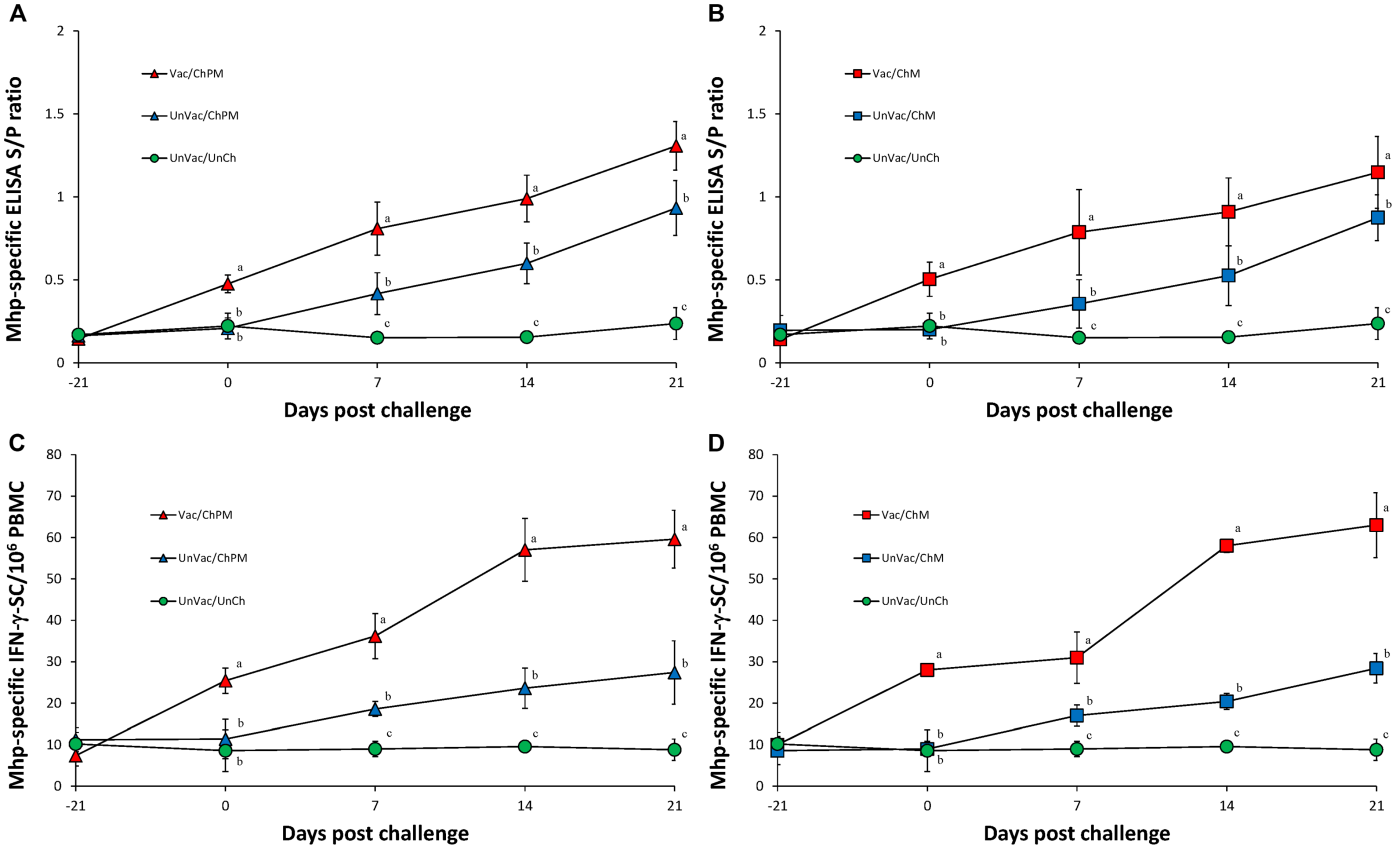
Immune responses against *Mycoplasma hyopneumoniae*. **(A)** Mean values of the anti-*M. hyopneumoniae* antibodies in PCV2d-and *M. hyopneumoniae*-challenged groups. **(B)** Mean values of the anti-*M. hyopneumoniae* antibodies in *M. hyopneumoniae*-challenged groups. **(C)** Frequency of *M. hyopneumoniae*-specific interferon-γ secreting cells (IFN-γ-SC) in PCV2d-and *M. hyopneumoniae*-challenged groups. **(D)** Frequency of *M. hyopneumoniae*-specific interferon-γ secreting cells (IFN-γ-SC) in *M. hyopneumoniae*-challenged groups. Different letters within a sampling point mean statistically significant differences (*p* < 0.05).

Vaccination and challenge (Vac/ChPM and Vac/ChM groups) resulted in significantly (*p* < 0.05) higher anti-*M. hyopneumoniae* antibody levels and *M. hyopneumoniae*-specific IFN-γ-SC levels as measured in Vac/ChPM and Vac/ChM group compared to the unvaccinated-challenged pigs (UnVac/ChPM and UnVac/ChM groups) at 0, 7, 14, and 21 dpc (Figure 5). Anti-*M. hyopneumoniae* antibody levels and *M. hyopneumoniae*-specific IFN-γ-SC were not detected in the unvaccinated-unchallenged pigs from the negative control (UnVac/UnCh) group throughout the entire experiment.

### Lung and lymphoid lesions

Vaccination and challenge (Vac/ChPM and Vac/ChM groups) resulted in significantly (*p* < 0.05) lower macroscopic and microscopic lung lesion scores compared to unvaccinated-challenged pigs (UnVac/ChPM and UnVac/ChM groups) at 21 dpc. Macroscopic and microscopic lung lesions were not observed in the unvaccinated-unchallenged pigs from the negative control (UnVac/UnCh) group (Table 2).

**Table 2:** Pathological outcomes (mean ± SD) in different groups at 21 days post challenge.

Group	Gross lung lesions (%)	Histopathology		PCV2 antigen in lymph node (+cell/0.25 mm^2^)
		Mycoplasmal lung lesions (score 0–6)	Lymph node lesions (score 0–5)	
Vac/ChPM	10.5 ± 7.16^a^	2.56 ± 0.38^a^	1.96 ± 0.64^a^	10.27 ± 2.56^a^
UnVac/ChPM	36.5 ± 18.17^b^	4.04 ± 0.84^b^	4.52 ± 0.46^b^	40.27 ± 12.94^b^
UnVac/UnCh	0 ± 0^c^	0 ± 0^c^	0 ± 0^c^	0 ± 0^c^
				
Vac/ChP	NT	NT	1.48 ± 0.41^a^	8.80 ± 1.82^a^
UnVac/ChP	NT	NT	2.44 ± 0.22^b^	20.40 ± 11.25^b^
UnVac/UnCh	NT	NT	0 ± 0^c^	0 ± 0^c^
				
Vac/ChM	9.0 ± 7.42^a^	2.28 ± 0.94^a^	NT	NT
UnVac/ChM	33.5 ± 6.98^b^	3.68 ± 0.64^b^	NT	NT
UnVac/UnCh	0 ± 0^c^	0 ± 0^c^	NT	NT

NT, not tested.

Different letters mean statistically significant differences (*p* < 0.05).

Vaccination and challenge (Vac/ChPM and Vac/ChP groups) resulted in significantly (*p* < 0.05) lower lymphoid lesion scores compared to unvaccinated-challenged pigs (UnVac/ChPM and UnVac/ChP groups) at 21 dpc. Lymphoid lesions were not observed in the unvaccinated-unchallenged pigs from the negative control (UnVac/UnCh) group (Table 2).

### PCV2 antigen in lymph nodes

Vaccination and challenge (Vac/ChPM and Vac/ChP groups) significantly (*p* < 0.05) lowered the number of PCV2 antigen positive-cells in pigs lymph nodes compared to unvaccinated-challenged pigs (UnVac/ChPM and UnVac/ChP groups) at 21 dpc. PCV2 antigen was not detected in the lymph nodes of the unvaccinated-unchallenged pigs from the negative control (UnVac/UnCh) group (Table 2).

## Discussion

A new bivalent vaccine containing PCV2d and *M. hyopneumoniae* provided good protection against an experimental dual PCV2d and *M. hyopneumoniae* challenge as observed by improved growth performance in vaccinated-challenged pigs (Vac/ChPM and Vac/ChM groups) compared to unvaccinated-challenged pigs. Unlike the other vaccinated-challenged groups, pigs vaccinated and then challenged with only PCV2d (Vac/ChP) did not significantly differ in growth performance from their unvaccinated-challenged counterparts (UnVac/ChP). As previously demonstrated, PCV2d alone is not enough to induce PCVAD experimentally, despite the fact that PCV2d is the primary causative agent of PCVAD (36).

Clinical outcomes of PCVAD are heavily dependent on PCV2 replication. High levels of PCV2 viremia led to clinical PCVAD with high mortality rates while low levels of PCV2 viremia led to a less obvious subclinical disease and reduced growth performance (37). This evaluation on PCV2 viremia is the primary indicator in the evaluation of PCV2 vaccine efficacy. The evaluated bivalent vaccine containing PCV2d and *M. hyopneumoniae* was able to significantly reduce PCV2d blood-viral load. Therefore, neutralizing antibodies against PCV2 and PCV2-specific IFN-γ-SC are considered protective immune responses as they play a key role in PCV2 clearance from both the blood and lymph nodes in swine as previous studies (38–42).

In our study, PCV2d-based bivalent vaccine elicits neutralizing antibodies (titer ≥ 2.5 log_2_) and IFN-γ-SC (frequency ≥ 30 × 10^6^ PBMC) at the time of challenge, increases gradually, and reaches the peak neutralizing antibodies (titer ≥ 6.5 log_2_) and IFN-γ-SC (frequency ≥ 108 × 10^6^ PBMC) at 21 dpc. On the other hands, unvaccinated pigs challenged with PCV2d (UnVac/ChPM and UnVac/ChP groups) cannot elicit the neutralizing antibodies and IFN-γ-SC at the time of challenge, and reach the low levels of neutralizing antibodies (titer ≥ 2 log_2_) and IFN-γ-SC (frequency ≥ 55 × 10^6^ PBMC) at 21 dpc. Therefore, differences in the levels of neutralizing antibodies and IFN-γ-SC are due to the vaccination between vaccinated-challenged (Vac/ChPM and Vac/ChP) and unvaccinated-challenged (UnVac/ChPM and UnVac/ChP) groups.

Porcine circovirus-associated disease is also characterized by lymphoid lesions such as granulomatous inflammation and lymphoid depletions (3, 4). Lymphoid lesions were significantly reduced in vaccinated-challenged pigs. The evaluated bivalent vaccine containing PCV2d and *M. hyopneumoniae* reduced the amount of PCV2d blood and lymph viral load in both and reduced the severity of lymphoid lesions, all elicitations of protective immunity.

Although humoral immunity of *M. hyopneumoniae* has not been associated with protection (43), cell-mediated immunity has proven to play a role in the protection of pigs from *M. hyopneumoniae* infection (44). ELISpot assays confirmed that the evaluated PCV2d and *M. hyopneumoniae* bivalent vaccine was successful in inducing a measurable cellular immune response while simultaneously reducing *M. hyopneumoniae* laryngeal loads and mycoplasmal lung lesion severity. These results conclude that this bivalent vaccine proved to be efficacious in protecting pigs against *M. hyopneumoniae* infection.

Porcine circovirus 2 vaccination provided better overall protection against a homologous genotype rather than a heterologous genotype challenge (45–47). However, other studies show conflicting results (48, 49). Results of PCV2d cross-protection by PCV2a vaccination based on levels of PCV2 viremia and PCV2-associated lesions are inconsistent. Several studies determined that PCV2a-based vaccines may produce a cross immune response to PCV2d and provide cross-protection against PCV2d infection (29, 46, 49, 50). These findings were contradicted in an additional study, where the evaluated PCV2a-based vaccine could not provide sufficient cross-protection against PCV2d infection based on immune responses (51). Nevertheless, our results should be interpreted cautiously because this study does not include heterologous vaccination and challenge groups, e.g., PCV2a vaccine against PCV2d challenge or PCV2d vaccine against PCV2a challenge. Further studies are needed to compare PCV2a vaccine against PCV2d challenge to PCV2d vaccine against PCV2a challenge under the same experimental conditions.

A PCV2d strain has a greater epidemiological significance as PCV2d is currently the most geographically predominant strain, particularly in most Asian countries including China, Korea, Taiwan, Thailand, and Vietnam (7, 9–12). PCV2d is more virulent than the traditional PCV2a and PCV2b genotypes in pigs dual-infected with PRRSV or *M. hyopneumoniae* but not with PCV2 alone (13–16, 52). Therefore, a new bivalent vaccine containing PCV2d and *M. hyopneumoniae* may be useful candidate vaccine in swine herds to protect against PCV2d and *M. hyopneumoniae* infection.

## Data availability statement

The original contributions presented in the study are included in the article/supplementary material, further inquiries can be directed to the corresponding author.

## Ethics statement

The animal study was reviewed and approved by the Seoul National University Institutional Animal Care and Use, and Ethics Committee (SNU-210308-2-1).

## Author contributions

SH: performance of the experimental trials and data analysis and writing of the manuscript. JS and TO: preparation of the inoculum and lab analysis. CK: making of the vaccine, design of the study, and writing of the manuscript. B-JS: making of the vaccine, design of the study, and writing of the manuscript. CC: development of protocol, design of the study, review of the final manuscript, and approval for publication. All authors contributed to the article and approved the submitted version.

## Funding

Korea Institute of Planning and Evaluation for Technology in Food, Agriculture and Forestry (IPET) through Technology Commercialization Support Program, funded by Ministry of Agriculture, Food and Rural Affairs (MAFRA; Grant no. 821032-03) and by the BK 21 FOUR Future Veterinary Medicine Leading Education and Research Center (Grant no. A0449-20200100).

## Conflict of interest

CK and B-JS were employed by WOOGENE B&G CO., LTD.

The remaining authors declare that the research was conducted in the absence of any commercial or financial relationships that could be construed as a potential conflict of interest.

## Publisher’s note

All claims expressed in this article are solely those of the authors and do not necessarily represent those of their affiliated organizations, or those of the publisher, the editors and the reviewers. Any product that may be evaluated in this article, or claim that may be made by its manufacturer, is not guaranteed or endorsed by the publisher.

## Footnotes

^1^ http://imagej.nih.gov/ij/download.html
